# Potential use of a diluted high-relaxivity gadolinium-based intra-articular contrast agent for magnetic resonance arthrography: an in-vitro study

**DOI:** 10.1186/s12880-019-0387-4

**Published:** 2019-10-25

**Authors:** Carmelo Messina, Domenico Albano, Davide Orlandi, Vito Chianca, Angelo Corazza, Federica Ferrari, Salvatore Gitto, Luca Maria Sconfienza

**Affiliations:** 1 IRCCS Istituto Ortopedico Galeazzi, Via R. Galeazzi 4, 20161 Milan, Italy; 2 Dipartimento di Scienze Biomediche per la Salute, Università degli Studi di Milano, Via Pascal 36, 20100 Milan, Italy; 3 Sezione di Scienze Radiologiche, Dipartimento di Biomedicina, Neuroscienze e Diagnostica Avanzata, Università degli Studi di Palermo, Via del Vespro 127, 90127 Palermo, Italy; 4 Dipartimento di Radiologia, Ospedale Evangelico Internazionale, Piazzale Gianasso 1, 16129 Genoa, Italy; 5 Scuola di Specializzazione in Radiodiagnostica, Università degli Studi di Milano, Via Festa del Perdono 7, 20122 Milan, Italy

**Keywords:** Magnetic resonance arthrography, Gadobenate dimeglumine, Gadopentetate dimeglumine, In vitro study

## Abstract

**Background:**

Magnetic resonance arthrography (MRA) requires intra-articular injection of gadolinium-based diluted paramagnetic contrast material. To our knowledge, gadobenate dimeglumine (Gd-BOPTA) has never been used for intra-articular applications. Our aim was to test in vitro different concentrations of Gd-BOPTA to be potentially used to perform MRA.

**Methods:**

Gd-BOPTA was diluted in saline (NaCl 0.9%) to achieve different concentrations (4 mmol/l; 2 mmol/l; 1 mmol/l; 0.67 mmol/l; 0.5 mmol/l). Six sets of five sterile pipes were prepared with 5 ml of each solution, five sets added with 0.5 ml of fresh synovial fluid. Two separate pipes were prepared with 5 ml of gadopentetate dimeglumine (Gd-DTPA) at 2 mmol/l, one pipe added with 0.5 ml of synovial fluid. Pipes were imaged using a T1-weighted sequence at 1.5 T. For each pipe, signal intensity (SI) in arbitrary units (au) was measured.

**Results:**

SI reproducibility range was 86–99%. Mean Gd-BOPTA SI in pipes containing synovial fluid increased from 1236 ± 8au (0.5 mmol/l) up to 1610 ± 44au (1 mmol/l) and down to 1405 ± 33au (4 mmol/l). Mean Gd-BOPTA SI in pipes without synovial fluid increased from 1184 ± 29au (0.5 mmol/l) up to 1530 ± 38au (1 mmol/l), and down to 1347 ± 39au (4 mmol/l). SI of pipes without synovial fluid was lower than that of pipes with synovial fluid for both Gd-BOPTA and Gd-DTPA (*P* ≤ 0.002). Regarding pipes with synovial fluid, mean Gd-DTPA SI at 2 mmol/l was 1246 ± 27au. Compared with Gd-BOPTA, SI was not different at 0.5 mmol/l (− 0.2%, *P* = 0.587) while it was higher (*P* < 0.001) at all other concentrations (range + 13.3%[4 mmol/l] − + 28.3%[1 mmol/l]). Regarding pipes without synovial fluid, mean Gd-DTPA SI at 2 mmol/l was 1275 ± 56au. Compared with Gd-BOPTA, SI was lower at 0.5 mmol/l (− 6.8%,*P* < 0.001), while it was higher (P < 0.001) at all other concentrations (range + 6.1%[4 mmol/l] − + 19.6% [1 mmol/l]).

**Conclusions:**

In vitro, Gd-BOPTA at 1 mmol/ had a + 28% SI increase in comparison to Gd-DTPA 2 mmol/l. SI similar to Gd-DTPA can be obtained using one fourth concentration of Gd-BOPTA.

## Background

Magnetic resonance imaging (MRI) has become the most important diagnostic tool for the assessment of joint disorders throughout the body, allowing for detecting abnormalities of the joint space, cartilage, fibrocartilages, tendons, ligaments, and synovial tissue [1]. In this setting, a further advanced tool is represented by MR arthrography (MRA), which is mainly used to evaluate young and less young patients and has been demonstrated to be superior in detecting a number of intra-articular joint abnormalities compared to conventional MRI [2, 3].

MRA requires intra-articular injection of gadolinium-based diluted paramagnetic contrast material [1, 4]. Contrast solution is injected until the capsule distends [5]. Traditionally, paramagnetic contrast solution was prepared manually, diluting a certain amount of gadolinium-based contrast agent into saline solution to obtain an approximate concentration of 2 mmol/l [5]. A recent survey showed this approach is still used in about 60% of institutions [6]. On the other hand, two commercial preparations are available as pre-filled syringes ready to be injected. These solutions are based on gadoteric acid (Gd-DOTA, 2.5 mmol/l; Dotarem, Guerbet, France) or on gadopentetate dimeglumine (Gd-DTPA, 2 mmol/l; Magnevist, Bayer, Germany). These two contrast agents have different molecular structures and properties but have similar R1-relaxivity values of approximately 4.2–5.3 l/mmols^− 1^ at 1.5 T and produce similar contrast enhancement when administered at the same dose [7]. Gadobenate dimeglumine (Gd-BOPTA, MultiHance, Bracco Imaging SpA, Italy) is a gadolinium-based contrast agent which has unique features compared to other agents, including very high R1-relaxivity thanks to a transient weak binding to blood proteins and is mostly used for liver imaging [8].

To our knowledge, Gd-BOPTA has never been used for intra-articular applications.

The purpose of our study was to test in vitro different concentrations of Gd-BOPTA to be potentially used for MRA.

## Methods

### Preparation of pipes

**Fig. 1:**
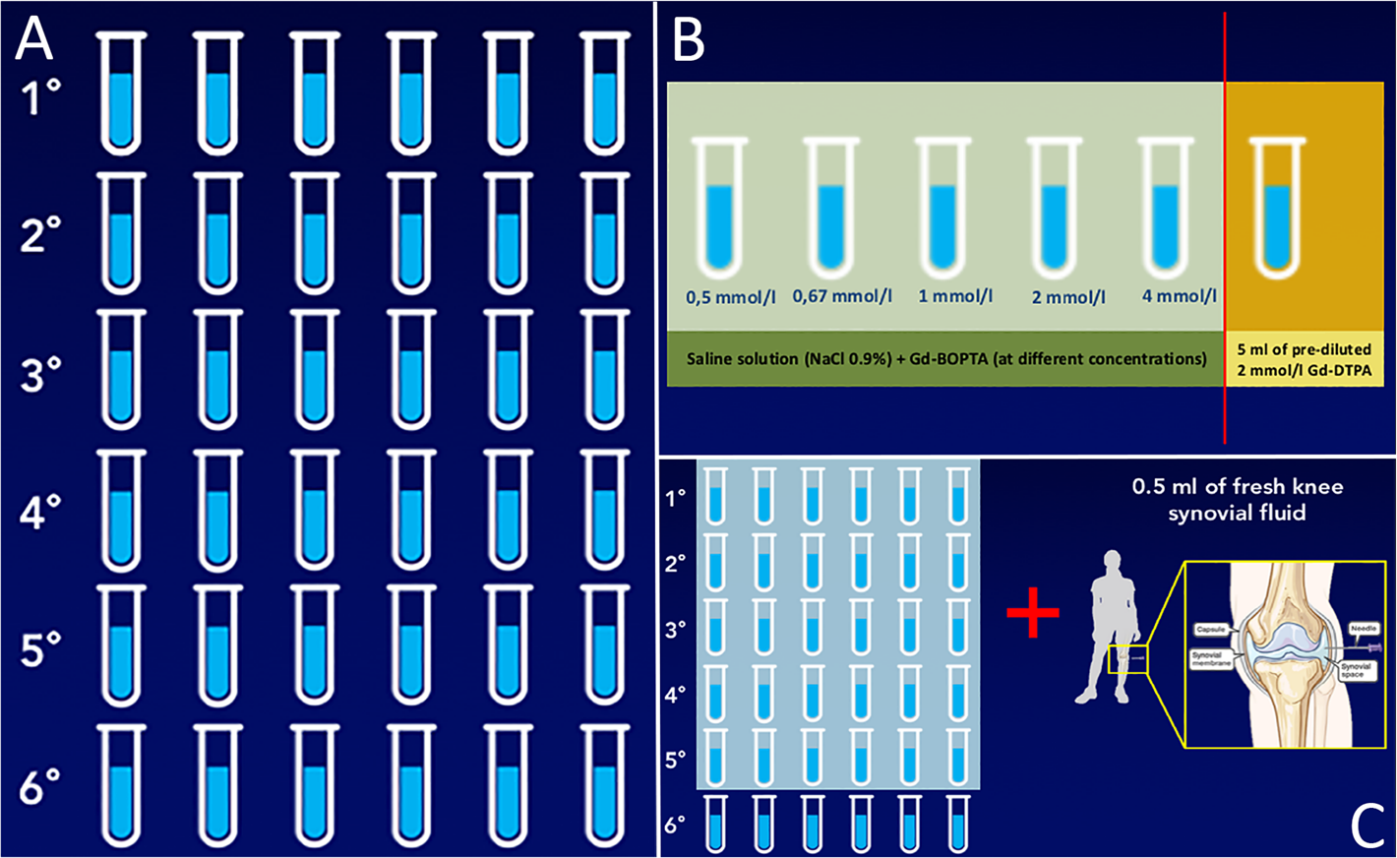
Six sets of six sterile pipes were prepared at different concentrations, 36 pipes overall (**a**). For all sets, Gd-BOPTA and saline solution (NaCl 0.9%) were mixed into five pipes in order to achieve a concentration of 4 mmol/l, 2 mmol/l, 1 mmol/l, 0.67 mmol/l, and 0.5 mmol/l; the sixth pipe was filled with 5 ml of 2 mmol/l pre-diluted Gd-DTPA and served as a reference (**b**). To simulate the environment of a joint, five sets of pipes were added with 0.5 ml of fresh synovial fluid withdrawn from the knees of five patients presenting to our department for intra-articular injections (**c**)

Six sets of six sterile pipes were prepared at different concentrations, 36 pipes overall (Fig. 1a). For all sets, Gd-BOPTA and saline solution (NaCl 0.9%) were mixed into five pipes in order to achieve a concentration of 4 mmol/l, 2 mmol/l, 1 mmol/l, 0.67 mmol/l, and 0.5 mmol/l; the sixth pipe was filled with 5 ml of 2 mmol/l pre-diluted Gd-DTPA and served as a reference (Fig. 1b). We used Gd-DTPA as reference standard since it is one of the most commonly used contrast agents in pre-diluted syringes for MRA examinations and it is also that used at our Institution.

To simulate the environment of a joint, five sets of pipes prepared as reported above were added with 0.5 ml of fresh synovial fluid withdrawn from the knees of five patients presenting to our department for intra-articular injections (three males, two females; mean age 68 ± 8 years) (Fig. 1c). These patients were all affected by degenerative osteoarthritis grade II according to Kellgren-Lawrence [9].

### Imaging protocol and statistical analysis

All six sets of pipes were imaged independently using an axial T1-weighted sequence (TE = 9.8 ms, TR = 678 ms, slice thickness 3.5 mm, FOV 320 × 320 mm) at 1.5 T (Sonata Maestro Class, Siemens Medical Solution, Erlangen, Germany) using a 4-channel phased-array surface coil. The same sequence was repeated twice to test reproducibility. Fourteen slices per pipe were obtained.

For each image, signal intensity (SI) in arbitrary units (au) was measured by one observer using a 4-mm diameter region of interest (Horos 3.3.5, www.horosproject.info) placed approximately in the middle of the image. For each pipe, the mean and standard deviation (SD) of SI over the 14 regions of interest was calculated. To compare the SI of Gd-BOPTA and Gd-DTPA, data from all the pipes and all the measurements were averaged.

Reproducibility between SI of each pipe was tested using the Bland-Altman method. Differences of SI between pipes at the same concentration of the two sets (with or without synovial fluid) and between pipes containing Gd-BOPTA at different concentrations and Gd-DTPA were assessed using the Mann-Whitney *U* test. Differences of SI among the five sets of pipes containing Gd-BOPTA per each concentration were tested using the Kruskal-Wallis test. The SPSS program (v.24, IBM, NY) was used. *P*-value threshold was set at 0.003 for multiple comparison correction.

## Results

**Fig. 2:**
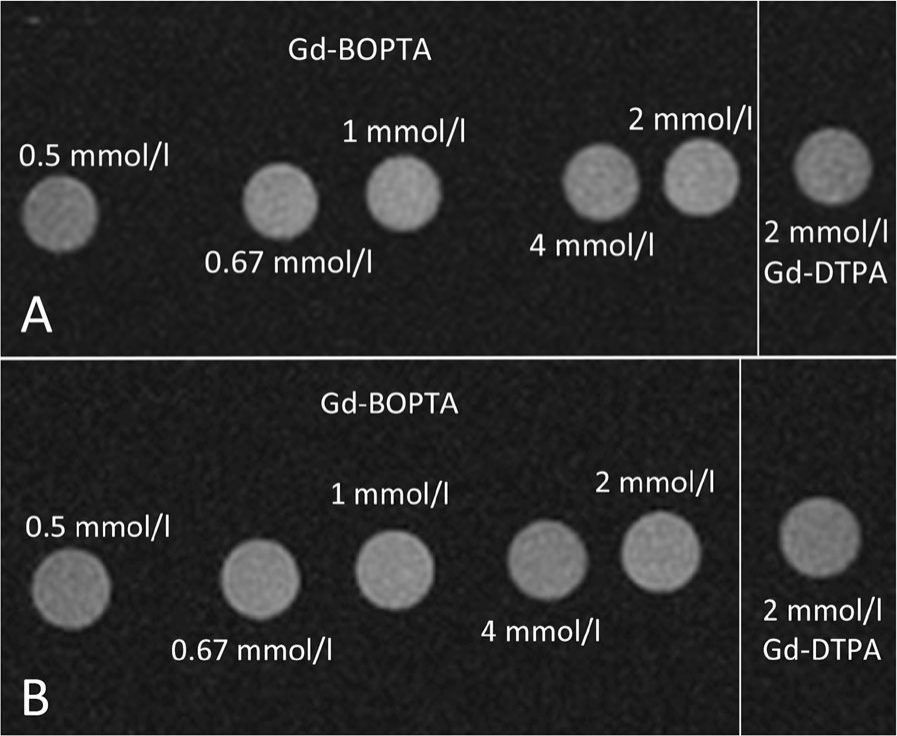
T1-weighted axial scan of two different sets of pipes containing different solutions of gadolinium-based contrast (**a**) with or (**b**) without addition of synovial fluid

Figure 2 shows T1-weighted axial scan of the two different sets of pipes containing different solutions of gadolinium-based contrast with and without addition of synovial fluid.

### Reproducibility

The reproducibility of SI measurement on the same pipe ranged from 86% (Gd-BOPTA 0.67 mmol/l without synovial fluid) to 99% (Gd-BOPTA 0.5 mmol/l, set #2 and set #3). Full data is reported in Table 1.

**Table 1:** Comparison of signal intensities of different solution concentrations of gadolinium-based contrast agents with or without addition of synovial fluid and their reproducibility

			Gd-BOPTA 0.5 mmol/l	Gd-BOPTA 0.67 mmol/l	Gd-BOPTA 1 mmol/l	Gd-BOPTA 2 mmol/l	Gd-BOPTA 4 mmol/l	Gd-DTPA 2 mmol/l	Saline
0.5 ml of synovial fluid	Set #1	Scan #1	1241 ± 19	1481 ± 50	1583 ± 34	1521 ± 29	1414 ± 34	1204 ± 59	403 ± 6
		Scan #2	1247 ± 13	1464 ± 60	1577 ± 35	1514 ± 26	1416 ± 37	1234 ± 47	403 ± 6
		Coefficient of repeatability	97%	90%	95%	95%	94%	89%	96%
	Set #2	Scan #1	1241 ± 8	1497 ± 49	1602 ± 52	1534 ± 39	1424 ± 37	1253 ± 50	391 ± 11
		Scan #2	1241 ± 7	1461 ± 62	1604 ± 34	1542 ± 32	1419 ± 36	1238 ± 61	398 ± 16
		Coefficient of repeatability	99%	90%	93%	94%	93%	87%	91%
	Set #3	Scan #1	1249 ± 13	1474 ± 45	1608 ± 37	1536 ± 35	1410 ± 32	1253 ± 37	398 ± 9
		Scan #2	1244 ± 21	1442 ± 54	1596 ± 34	1527 ± 29	1415 ± 32	1234 ± 40	400 ± 7
		Coefficient of repeatability	99%	90%	93%	94%	93%	87%	91%
	Set #4	Scan #1	1236 ± 8	1486 ± 61	1610 ± 44	1524 ± 32	1406 ± 38	1232 ± 46	409 ± 8
		Scan #2	1243 ± 17	1470 ± 46	1584 ± 37	1521 ± 32	1408 ± 28	1239 ± 53	409 ± 7
		Coefficient of repeatability	96%	90%	94%	93%	94%	90%	94%
	Set #5	Scan #1	1246 ± 19	1496 ± 49	1601 ± 38	1540 ± 26	1411 ± 34	1266 ± 55	404 ± 6
		Scan #2	1240 ± 14	1484 ± 44	1601 ± 27	1545 ± 27	1405 ± 33	1246 ± 46	403 ± 10
		Coefficient of repeatability	96%	91%	95%	95%	93%	91%	95%
	Differences between sets		0.714	0.285	0.213	0.013	0.411	0.165	0.000
No synovial fluid	Set #6	Scan #1	1184 ± 29	1441 ± 49	1530 ± 38	1478 ± 39	1347 ± 39	1292 ± 45	401 ± 5
		Scan #2	1191 ± 31	1383 ± 58	1520 ± 36	1454 ± 34	1358 ± 35	1257 ± 62	387 ± 10
		Coefficient of repeatability	94%	86%	93%	93%	94%	89%	92%

### SI of Gd-BOPTA pipes

The mean SI of Gd-BOPTA in pipes containing synovial fluid increased from 1236 ± 8 au at 0.50 mmol/l (set #4, scan #1) up to 1610 ± 44 au at 1.00 mmol/l (set #4, scan #1) and down to 1405 ± 33 au at 4.00 mmol/l (set #5, scan #2). SI within pipe sets was homogeneous, with no significant differences among them (*P* > 0.004).

The mean SI of Gd-BOPTA in pipes not containing synovial fluid showed an increase from 1184 ± 29 au at 0.50 mmol/l (set #6, scan #1) up to 1530 ± 38 au at 1.00 mmol/l (set #6, scan #1), and down to 1347 ± 39 au at 4.00 mmol/l (set #6, scan #1).

SI of pipes without synovial fluid was significantly lower than that of pipes with synovial fluid for both Gd-BOPTA and Gd-DTPA (*P* ≤ 0.002).

**Fig. 3:**
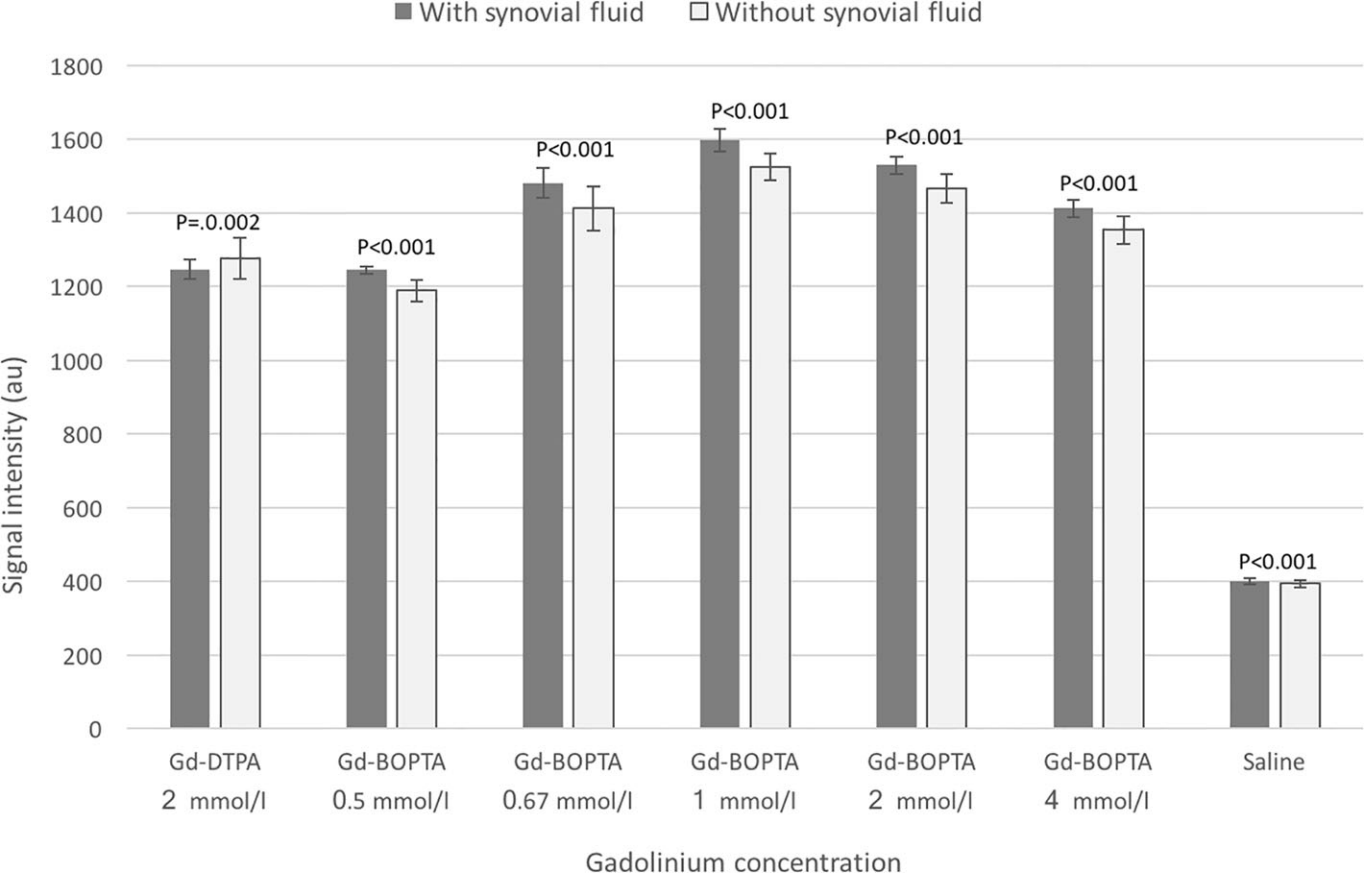
Graphical representation of signal intensity of different solutions of gadolinium-based contrast agents with or without addition of synovial fluid and saline solution. Signal intensity of pipes without synovial fluid was significantly lower than that of pipes with synovial fluid

Full data is reported in Table 1 and graphically represented in Fig. 3.

### Comparison between Gd-BOPTA and Gd-DTPA

Regarding pipes with synovial fluid, the mean SI of Gd-DTPA at 2 mmol/l was 1246 ± 27 au. In comparison with Gd-BOPTA, SI was not significantly different at 0.5 mmol/l (− 0.2%, *P* = 0.587) while it was significantly higher at all other concentrations (range + 13.3% at 4 mmol/l to 28.3% at 1 mmol/l, *P* < 0.001).

Regarding pipes without synovial fluid, the mean SI of Gd-DTPA at 2 mmol/l was 1275 ± 56 au. In comparison with Gd-BOPTA, SI was significantly lower at 0.5 mmol/l (− 6.8%, P < 0.001) while it was significantly higher at all other concentrations (range + 6.1% at 4 mmol/l to 19.6% at 1 mmol/l, P < 0.001). Full data is reported in Table 2.

**Table 2:** Comparison of signal intensities of different Gd-BOPTA concentrations to Gd-DTPA 2 mmol/l with or without addition of synovial fluid

			Gd-BOPTA 0.5 mmol/l	Gd-BOPTA 0.67 mmol/l	Gd-BOPTA 1 mmol/l	Gd-BOPTA 2 mmol/l	Gd-BOPTA 4 mmol/l	Saline
With synovial fluid	Gd-DTPA 2 mmol/l		1244 ± 9	1481 ± 41	1598 ± 30	1529 ± 24	1412 ± 23	401 ± 8
		1246 ± 27	−0.2%	18.9%	28.3%	22.7%	13.3%	−67.8%
			P = 0.587	P < 0.001	P < 0.001	P < 0.001	P < 0.001	P < 0.001
Without synovial fluid			1188 ± 29	1412 ± 60	1525 ± 36	1466 ± 38	1353 ± 37	394 ± 10
		1275 ± 56	−6.8%	10.7%	19.6%	15.0%	6.1%	−69.1%
			P < 0.001	P < 0.001	P < 0.001	P < 0.001	P < 0.001	P < 0.001

Note. - *P*-values and percentages refer to comparison between the corresponding concentration of Gd-BOPTA and Gd-DTPA

## Discussion

Our in vitro experience demonstrated that synovial fluid slightly but significantly increased SI of Gd-BOPTA. In pipes containing synovial fluid, Gd-BOPTA at 1 mmol/l had a + 28% SI increase in comparison to Gd-DTPA 2 mmol/l.

Gd-BOPTA has unique features that distinguish it from all other gadolinium-based contrast media. In particular, it has a dual route of elimination from the body that enables Gd-BOPTA to be used both as nonspecific agent and liver-specific agent [10]. Moreover, a higher R1-relaxivity compared to other gadolinum-based contrast agents permits lower overall doses to be used or provides increased SI enhancement at equivalent dose [7]. In fact, Gd-BOPTA has a weak and transient interaction with serum albumin which confers a partial blood-pool effect and a lower rate of contrast dilution compared to that of other gadolinium agents [11]. Indeed, Gd-BOPTA has been proven to be effective in brain [12], vascular [13], liver [14], and breast MRI [15], in which it can be used at lower doses compared to other gadolinium-based agents. At present, however, the use of Gd-BOPTA has been limited to the liver only, given the reported accumulation of Gd in patients’ tissues after repeated intravenous administrations [16, 17]. On the other hand, it should also be noted that a recent report showed how Gd does not accumulate in patients undergoing MRA [18].

Regarding MRA, the use of Gd-BOPTA has never been clearly reported before except for an animal study published in 2007 in which its effect on cartilage was tested [19]. However, in some studies dealing with MRA the type of contrast agent may be not reported [20]. Moreover, other studies used Gd-BOPTA to perform indirect MRA [21], an alternative imaging technique based on the premise that contrast material administered intravenously diffuses into the joint space, owing to high vascularization of the synovial membrane and lack of basement membrane in its capillaries. Compared with the direct technique, indirect MR arthrography has the main disadvantage of not allowing for capsular distension. On the other side, intravenous injection allows assessing disease activity in patients affected by inflammatory joint diseases [22]. At any rate, the use of direct MRA is continuously increasing, notwithstanding improved coils at 1.5 T and increased diffusion of 3 T systems.

Compared to 2 mmol/l solution of Gd-DTPA, SI of Gd-BOPTA was higher at all concentrations except 0.5 mmol/l. The highest SI was achieved at 1 mmol/l, while a similar SI was obtained at 0.5 mmol/l in pipe sets added with synovial fluid. In clinical practice, these differences may be negligible, implying that also a low concentration of gadolinium-based solution can be considered acceptable. The main practical result of these findings is that, in a simulated joint environment, the same SI of pre-diluted Gd-DTPA syringes can be obtained with up one fourth concentration of Gd-BOPTA. However, in vivo testing of this speculation should be warranted.

We acknowledge the limitations of an in vitro study in which joint environment has been reproduced only adding a small amount of synovial fluid to the contrast agent contained in sterile pipes. This implies that our data may be not directly applicable in vivo, as other factors (e.g., synovial tissue, inflammatory cytokines) may be implicated in the interaction with Gd-BOPTA. Also, body temperature may somewhat affect behavior of contrast agents in vivo, while our experiment was conducted at room temperature. Moreover, we used the synovial fluid withdrawn from the knees of patients with a mean age of 68 years and mild degenerative osteoarthritis. It is known that synovial fluid of elderly subjects may contain less glycoproteins and glycosaminoglycans. Thus, in younger subjects, in whom MRA is more likely to be performed, results may be somewhat different. We can speculate that a higher content of glycoproteins may further improve SI, although at present we do not have data to confirm such hypothesis. Finally, we did not assess separately T1 and T2 effects of the contrast media and our results are only valid for one specific parameter set TR and TE. Also, relative signal changes with noise correction should also be measured to assess reliable relaxivity values R1 and R2. However, our study was primarily aimed to measure SI, that is the most useful parameter that may affect clinical practice.

## Conclusions

In conclusion, in vitro, Gd-BOPTA at 1 mmol/ had a + 28% SI increase in comparison to Gd-DTPA 2 mmol/l. A SI similar to Gd-DTPA can be obtained using one fourth concentration of Gd-BOPTA. Further in vivo studies are warranted to confirm clinical applicability and value of our results.

## Data Availability

All data are fully available upon reasonable request. The corresponding author should be contacted if someone wants to request the data.

## Abbreviations

Gd-BOPTA: Gadobenate dimeglumine
Gd-DOTA: Gadoteric acid
Gd-DTPA: Gadopentetate dimeglumine
MRA: Magnetic resonance arthrography
MRI: Magnetic resonance imaging
